# Firefighters’ Clothing Contamination in Fires of Electric Vehicle Batteries and Photovoltaic Modules—Literature Review and Pilot Tests Results

**DOI:** 10.3390/ijerph191912442

**Published:** 2022-09-29

**Authors:** Ewelina Szmytke, Dorota Brzezińska, Waldemar Machnowski, Szymon Kokot

**Affiliations:** 1 Faculty of Process and Environmental Engineering, Łódź University of Technology, Wolczanska 213 Str., 90-924 Lodz, Poland; 2 Institute of Material Science of Textiles and Polymer Composites, Faculty of Material Technologies and Textile Design, Łódź University of Technology, Żeromskiego 116 Str., 90-924 Lodz, Poland; 3 cfbt.pl Foundation, Poranna 12 Str., 11-041 Olsztyn, Poland

**Keywords:** firefighter safety, firefighters, PV fire, electrical fire, car battery, electric vehicle, firefighters’ protective clothing, firefighters’ clothing cleaning, liquid CO_2_ firefighters’ clothing decontamination

## Abstract

The electric vehicle (EV) market, together with photovoltaic (PV) installations continues to develop at a pace. However, there are concerns that EV and PV installation fires may create more harmful substances than other types of fire. PV modules and car battery fires emit a range of carcinogenic and highly toxic compounds that are not yet fully understood and may pose a threat to firefighters’ health. This also raises the question of the impact on firefighters’ clothing and the safe handling and cleaning after such fires. This article presents a literature and standards review of the firefighters’ protective clothing maintenance and cleaning. It also contains test results showing that firefighters’ clothes accumulate harmful substances after fighting these types of fires. Pilot tests for the presence of polycyclic aromatic hydrocarbons (PAHs) and formaldehyde showed that levels exceeded limits in all clothing samples. For example, the cobalt level was 24 times higher than that considered safe in the test carried out with car battery fire. Although it is recognized that liquid carbon dioxide (LCO_2_) methods of cleaning may be more effective than traditional water washing, further research on cleaning efficiency for clothing containing substances emitted from car battery and PV modules fires is required.

## 1. Introduction

As a result of the global trend to develop and implement green technologies, many advances have been made in the energy sector. Two obvious examples are the growing number of photovoltaic (PV) installations in buildings [1] as well as car batteries used as electrical power for propulsion [2,3]. With such growth, it has been noticed that a significant number of fires have resulted from the failure of PV installations [4], as well as fires involving electric vehicles [5,6]. In such incidents, firefighters are exposed to smoke containing highly toxic, flammable, corrosive, and irritant substances [7,8].

Car battery failure or damage leads to dangerous gases release such as carbon monoxide (CO), hydrogen fluoride (HF), hydrogen cyanide (HCN), phosphorous oxyfluoride (POF_3_) that can pose a serious threat to those involved [5,9], and hydrogen (H_2_) or methane (CH_4_) that increase the development of fire, and could create jet flames, or even explosion [9,10,11,12,13]. The PV installation, in turn, contains a significant share of plastic materials, such as encapsulants, back sheet foils, junction boxes, and insulants of cables that, in the event of a fire, emit a mixture of gases, burning droplets, and solid soot particles mixed with different organic compounds, depending on the combustion effectiveness. The fires of PV installations produce smoke containing carbon monoxide (CO), carbon dioxide (CO_2_), carbon fluoride (CF), cadmium (Cd), acetic acid (CH_3_COOH), dimethyl butane (C_6_H_14_), and aliphatic compounds as well as different volatile organic compounds (VOCs) [14,15].

Due to the emissions mentioned above, the PV installations and batteries during combustion create a chemical risk to firefighters. The effects of toxicity on fighters’ health is greater when the toxic substances are trapped within the firefighters’ Personal Protective Clothing (PPC). The firefighters’ clothing must fulfill many important functions under fire conditions, such as resistance to water, heat, and fire, while providing the required level of work comfort and safety [16]. In addition, to protect against burns, the clothing should exhibit high mechanical performance properties, provide protection against liquid chemicals, and possess an optimal thermo-physiological comfort for the user. For this reason, firefighters’ clothing usually consists of four basic layers with different purposes: outer shell, moisture barrier membrane, thermal barrier layer, and inner liner [17,18]. Most of the materials used in the production of firefighters’ clothing are porous textiles made of fibers that are capable of absorbing and retaining the toxic gases and vapors produced at fires mentioned above. The multilayer structure of the firefighters’ clothing and the absorption properties of the fibers create obstacles to effective cleaning and removal of the harmful substances accumulated in the event of fire [19,20]. This may lead to different firefighters’ illnesses [21] as they are kept exposed to the substances trapped in and on the surface of the used PPC as well as in the fire station, or inside, and on the vehicles used [20,21,22,23,24]. VOCs, or PAHs, are found to have a longer-term effect as a result of repeated exposure to even very small amounts (chronic toxicity) and cause adverse effects on health as they accumulate and slowly develop cancer, cardiovascular, neurological diseases, etc. [25]. Therefore, it is highly recommended to clean the protective clothing effectively [26,27,28].

No information was found in the available literature on the criteria for determining the safe re-use of cleaned firefighter protective clothing and how to handle this clothing contaminated by smoke from PV modules or car battery fires. These issues are the subject of the literature review presented in this article. Although there are known reports on the toxic substances’ volume production during battery and PV module fires, there is no information on how much of them could be absorbed by the firefighters’ clothes. Also, the fire conditions that influence this phenomenon were not previously investigated. This article presents experimental results, which demonstrate the PPC contamination during car battery and PV fires in an open and closed space.

For clarity, a section dedicated to abbreviation and short forms used in the manuscript has been added at the end of the article.

## 2. Existing Guidelines and Practice in Firefighters’ PPC Maintenance

### 2.1. Standards of PPC Maintenance

**Figure 1:**
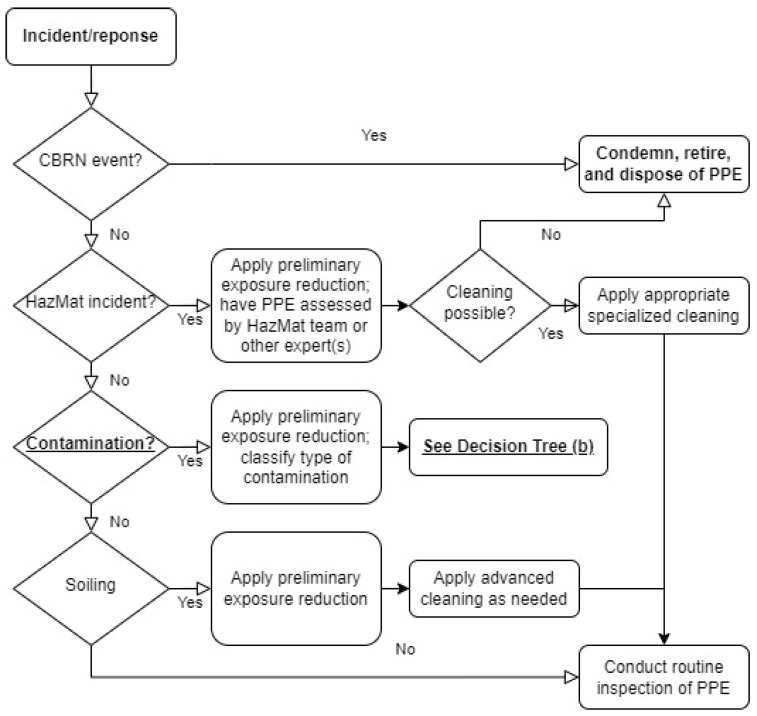
Approach for deciding handling, cleaning, and disposal of ensemble elements according to NFPA 1851 [29].

**Figure 2:**
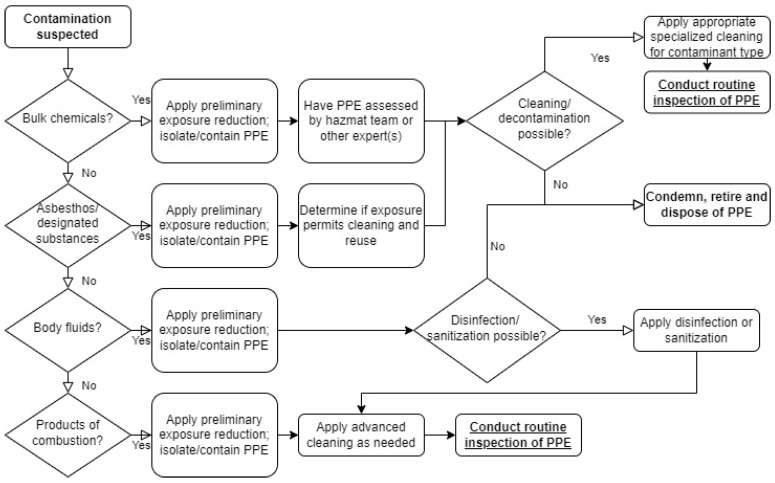
Approach for addressing specific types of contamination according to NFPA 1851 [29].

As it comes to guidelines for proper PPC requirements and maintenance, there have been several updated standards, such as: NFPA 1851 [29], BS 8617: 2019 [30], EN 469: 2020 [31], EN 13911: 2017 [32], EN ISO 15384: 2018/AMD 1:2021 [33] and ISO 23616: 2022 [34]. The following are their most important aspects relative to the article’s subject:

- NFPA 1851: 2020 Standard on Selection, Care, and Maintenance of Protective Ensembles for Structural Fire Fighting and Proximity Fire Fighting [29]—when it comes to Personal Protective Equipment (PPE) cleaning, this standard includes two tree diagrams to assist in the decision making on how to handle, clean, and dispose of PPE. PPE is a wider term that also includes PPC. The first decision tree (Figure 1) concerns general guidance, while the second decision tree (Figure 2), is specific to different types of contamination.
  As shown in Figure 1, the general decision path includes the type of event where PPE was used, especially if this was a chemical, biological, radiological, or nuclear (CBRN) event, and what classified it for retiring. The next step is an analysis of where the Hazardous Materials (HazMat) were detected. This determines whether cleaning is possible or not. The PPE classified for cleaning should be processed in a specialized service and be subject to a routine inspection. The third decision concerns other types of contamination. It suggests PPE should be secured to reduce the firefighters’ exposure to harmful substances, and the type of contamination should be classified as shown in Figure 2.
  The procedures presented in Figure 2 concern the following decision path when PPE is suspected to be contaminated. It consists of the verification of the presence of bulk chemicals and asbestos followed by appropriate action recommendations.
  The NFPA 1851 standard also provides an advanced PPE cleaning twice a year frequency recommendations, with one annual advanced inspection at least. New broad guidelines were also added for cleaning and sanitizing protective coats and pants, suggesting verification once each two years. However, an advanced inspection has been indicated at least annually (as opposed to year three in-service) or whenever a routine inspection may suggest potential damage. Considering the contaminants from PV installation and car battery fires, according to the NFPA 1851 decision tree, this type of incident should be treated as contamination with products of combustion, which is a very wide group of fires, including residential building fires, etc. However, it may be assumed that the types of substances in smoke may vary significantly. Although the verification of the cleaning procedure is mentioned in NFPA 1851, this means that the service provider is obliged to send contaminated samples for testing after advanced cleaning, and the result must provide at least 50% efficiency for removal of the average of all surrogate heavy metal contamination and semi-volatile organic compounds. Maximum level of contamination is not mentioned. The standard only indicated the fraction that needs to be disposed of.
- BS 8617: 2019 Personal protective equipment for firefighters—Cleaning, maintenance, and repair [30]—this standard establishes guidance for cleaning, maintenance, and repairing of different elements of firefighters’ PPE, in order to reduce the potential health and safety risks resulting from a poorly maintained, contaminated or damaged equipment. It includes inspection, testing, cleaning, decontamination, drying, repairs, replacement, retirement/disposal, recording, storage, and transportation, but without any detailed recommendations.
- EN 469: 2020 Protective clothing for firefighters—Performance requirements for protective clothing for firefighting [31]—this standard contains minimum performance requirements for protective clothing intended for use during firefighting operations, including construction, protection against heat and flame, mechanical and chemical properties, in terms of comfort of use and visibility, distinguishing between actions carried out outdoors and in buildings, in terms of protection against heat and flame.
- EN 13911: 2017 Protective clothing for firefighters—Requirements and test methods for fire hoods for firefighters [32]—this standard presents minimum safety requirements and test methods for a firefighters’ balaclava to be worn during rescue and firefighting operations to protect against the effects of heat and fire.
- EN ISO 15384: 2018/AMD 1: 2021 Protective clothing for firefighters—Laboratory test methods and performance requirements for wildland firefighting clothing + Amendment 1 [33]—this standard contains test methods and minimum performance requirements for PPC designed to protect the users’ body, excluding the head, hands, and feet, which is used in open-air firefighting and related activities. Wildland firefighting clothing refers to clothing which is used in open-air firefighting. However, this standard does not describe PPC maintenance procedures.
- ISO 23616: 2022 Cleaning, Inspection and Repair of Firefighters’ PPE [34]—this standard refers to requirements, guidance, and recommendations for PPE cleaning, inspection, and repairing. This standard excludes information concerning chemical protective clothing as well as CBRN protective clothing handling, apart from the information that once the PPE is used in a CBRN event, it should be secured and disposed of properly. It suggest periodical PPC washing and careful mechanical inspection of the clothing after this.

The existing recommendations presented in the standards mentioned do not clearly demonstrate the proper cleaning/disposal procedures for contaminated firefighters’ clothing. Moreover, it was observed that the cleaning and decontamination processes are not sufficiently distinguished in the presented standards, so the additional literature review in this field is submitted in Section 2.2.

### 2.2. Cleaning and Decontamination Processes

Cleaning and decontamination are often considered to mean the same process. However, there is a significant difference between them. Cleaning is a more general term and includes decontamination as a more specific process [35]. When decontamination takes place, acceptable criteria should be specified. If such a guideline is met, the PPC may be considered safe for use [36,37]. Today, firefighters’ clothing is not subject to decontamination in this sense, and the level of impurities contained is not measured after cleaning. The clothes are most often assessed visually only. As mentioned before, even NFPA 1851 [29] and ISO 23616: 2022 [34] do not specify the maximum limits of toxic substances in PPC. It can be considered that inspection described in the reviewed standards is a subjective assessment with no confirmation in actual measurements that could guarantee firefighters’ clothing safe for reuse. Standards included in the literature review do not specify what the regular and advanced cleaning methods are, but the assumption of the authors of this article is that water cleaning using detergents and using different wash programs, is the most common form of cleaning. As this literature review has stated other potentially more effective contamination removal methods, such as LCO_2_, exist. Nevertheless, this new technology still needs further research. In addition, sufficient resources and infrastructure that would enable wide implementation of this technology are not yet in place.

As literature review shows, guidelines already exist that specify the maximum limits of harmful substances in textiles in terms of health safety. In 2021, a new edition of Standard 100 by OEKO-TEX^®^ was released concerning textile product safety [37]. OEKO-TEX^®^ is a registered trademark that represents the product labels and company certifications issued by the International Association for Research and Testing for textiles and leather ecology. The organization with headquarters in Zürich (Switzerland) was founded in 1992. Founding members were the German Hohenstein Institute and Institut fuer Oekologie, Technik und Innovation GmbH (OETI). Currently, the Oeko-Tex Association includes 18 neutral test and research institutes with contact offices with a global reach.

Annex 4 and 6 to this Standard specifies values of different contaminants that may not be exceeded in order to obtain the OEKO-TEX^®^ certificate and be considered safe for use in direct contact with skin or with no direct contact with skin—depending on the purpose of the material. Examples of these acceptable limits are shown in Table 1.

Another example of specified acceptable limits of harmful substances that may be present in items that have contact with human is the Ausschuss für Produktsicherheit (AfPS) guideline called the GS specification [38]. AfPS is the German Product Safety Commission. This document concerns all the consumer products that are to be introduced to the market and refers to the maximum PAH level. As the article concerns firefighters’ clothing, Table 2 shows the levels of PAHs specified for adults only.

Restrictions in placing products on the market and the use of certain dangerous substances (such as specified PAHs) mentioned before were also implemented at the European level as the European Chemicals Agency (ECHA) in the regulation of the European Union to improve the protection of human health and the environment, called REACH (in Annex VII)t [39].

Nevertheless, none of the mentioned specifications have a direct link to specific requirements on firefighters’ clothing handling after contamination and cleaning.

### 2.3. Firefighters’ Protective Clothing Handling: Methods and Practices

Currently, the type of cleaning of firefighters’ protective clothing considered the best practice is washing it in water with detergents. For this purpose, specialized washing machines are provided at fire stations, or the service is outsourced [35]. However, studies on the effectiveness of water laundering show relatively low efficiency of this method [40]. Despite the use of intensive water washing (at the temperature of 60 °C) in specialist appliances, a washing efficiency of more than 40% was not achieved. PAHs and other harmful substance levels in the PPC exceeded the specified maximum limits of the AfPS for PAHs [38]. The researchers who evaluated the efficiency of the water cleaning processes [40] summarized that there are ways that may improve it, primarily by reducing the number of clothes that are cleaned simultaneously. Decreasing the storage period of the contaminated PPC before washing may also have a positive influence. Additionally, ozone treatment or LCO_2_ cleaning methods were recommended for further research.

A study on the evaluation of an ozone chamber as a routine method of decontamination of firefighters’ clothing was carried out in Madrid, Spain [41]. This study indicated the limited effectiveness of such a process. It was observed that large amounts of PAHs remained in the samples even after one hour of ozone treatment. Partial chemical degradation of PAHs took place. However, the remaining concentration of PAHs, and equally or more toxic oxygenated PAH compounds created in the process alerted the authors of the study to potential health risks to firefighters.

The LCO_2_ cleaning method seems to be more efficient, as tests carried out by The University of Leuven show [42]. The tests were performed with three groups of firefighters. One group wore contaminated PPC (without washing), the second group wore contaminated PPC cleaned by industrial laundry (according to ISO 15797-2 [43]), and the last group wore contaminated PPC cleaned with LCO_2_. In the experiment, blood tests were performed in order to detect the presence of harmful substances. The results showed that firefighters who wore the most contaminated clothes (without washing) had the highest concentrations of toxic substances in their blood. The second highest obtained results were seen in the group of firefighters whose clothes were washed in an industrial water laundry. Finally, in the group of firefighters whose clothes were decontaminated with LCO_2_, no significant increase in toxic substances in blood was observed.

However, more research is needed to verify whether the LCO_2_ method is as effective in removing different harmful substances from firefighters’ clothing, including those emitted from car battery and solar power module fires. This article examines the types of contamination of firefighters’ clothing from PV modules and car battery fires.

## 3. Results of Firefighters’ Clothing Contamination from PV Module and Car Battery Fires

PPC contamination experiments during car battery and PV module fires were conducted in Olsztyn, Poland, on 18 September 2021. New firefighters’ clothing was exposed to products of combustion in various scenarios.

The experiments included three pilot full-scale fire tests described in Table 3.

The PV modules and the car battery were located over an Oriented Strand Board (OSB) and wood pile on a steel construction in all tests. The pile dimensions were 40 × 65 × 55 cm. There were two sizes of OSB fragments that made up the pile. Pinewood pieces and kindling cubes were also used, as shown in Table 4. The pinewoods’ moisture was about 16%. The pile was ignited by a gas jet in all three tests.

In Test 1, the firefighters’ clothing was located 0.5 m away from the PV module placed on steel construction. This test was carried out in the open space. The ambient temperature was 9.6 °C, and the humidity varied between 74 and 83%. There was no wind observed. The airflow was arranged by a portable regulated fan, which directed smoke to the firefighters’ clothing on a mannequin standing next to the burning photovoltaic module. The fan was located 3.2 m away from the PV module. The air velocity was measured about 1.5 m away from the PV module, and the portable fan (between those items) was up to 9.16 m/s.

**Figure 3:**
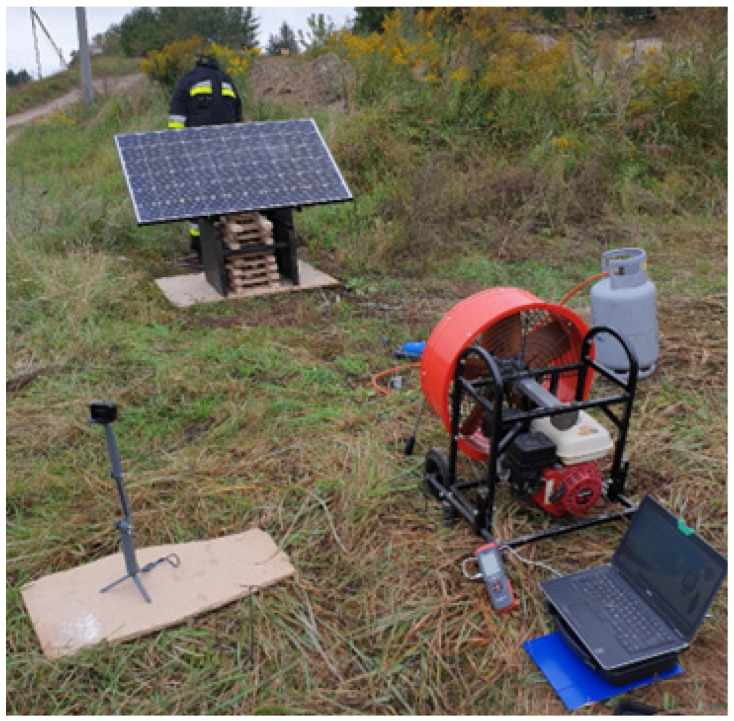
Scenario Test 1 with a PV module fire in an open space. The photograph shows the test stand and the fan that directed the airflow to the PPC.

The PV module was located over an OSB pile to simulate a roof fire underneath, as presented in Figure 3. Test 1 lasted 7 min.

The PV module fire experiment was repeated inside a closed training container (Test 2). The container was 11.82 m long, 2.45 m wide, and 2.50 m high. The container had many openings in different locations in order to provide the airflow needed in a test scenario. One of them was open 1 m away from the PV module to provide air supply. The size of this air opening was 1.04 m × 0.53 m. This was also the location where the ambient temperature, humidity, and air velocity were measured during Tests 2 and 3. The air velocity at the supply point was up to 0.48 m/s.

**Figure 4:**
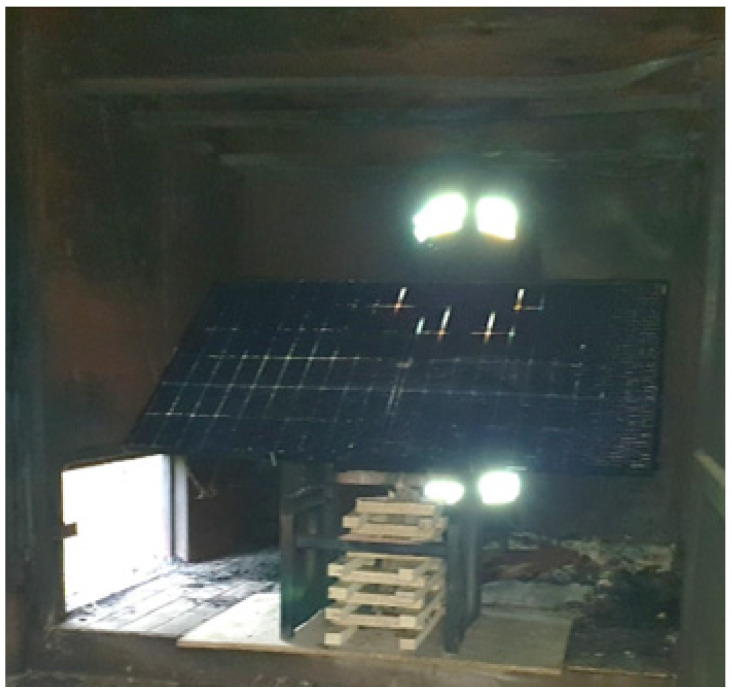
Scenario Test 2 with a PV module fire in a closed space.

In this experiment, another new set of firefighters’ clothing was used for contamination, placed 0.5 m away from the PV module over the fire source on a steel construction (Figure 4). Test 2 also lasted 7 min.

**Figure 5:**
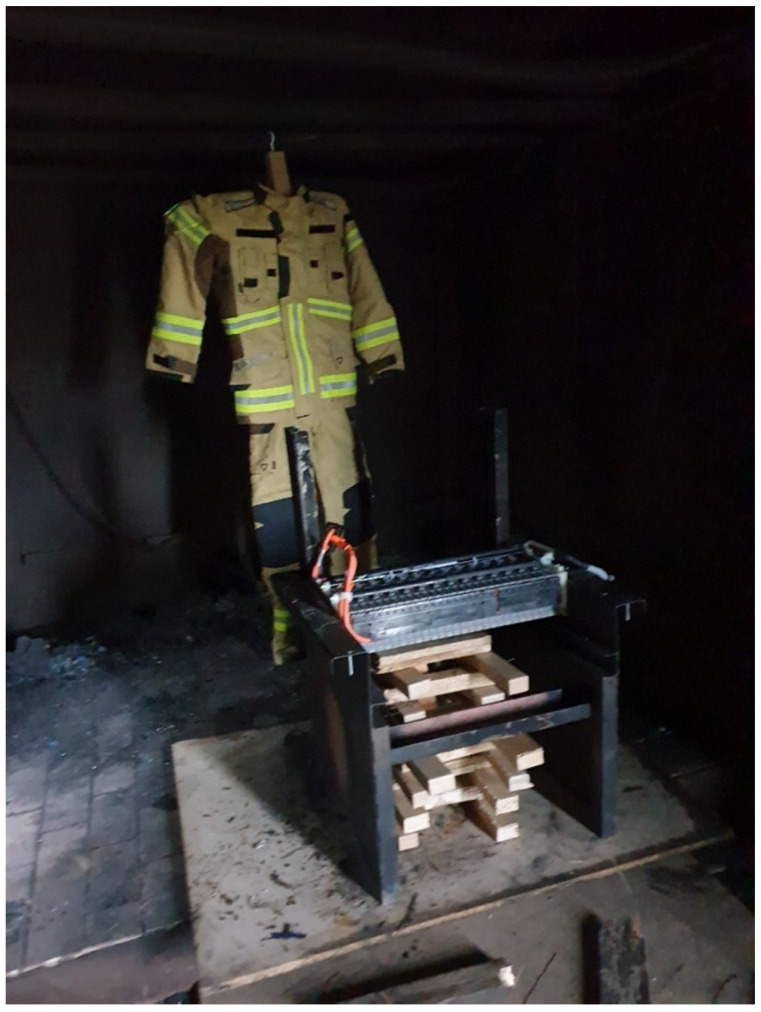
Scenario Test 3 with a car battery in a closed space.

The third experiment (Test 3) was conducted with a car battery located on a steel construction in the same closed container as previously. In this test, a Ballyclare firefighters’ clothing was located 0.5 m away from the burning car battery on a steel construction as in the previous tests (Figure 5). The same air supply was opened 1 m away from the car battery.

The air velocity at the Test 3 supply point was up to 1.59 m/s in the last minute of the test. Nevertheless, it is worth mentioning that the air velocity at the air supply was 0 m/s since the Test 3 started for 9 min. Test 3 lasted 14 min, as car battery ignition was observed significantly later than PV module ignition in Tests 1 and 2.

The samples taken for the experiment came from contaminated jackets. After the pilot fire tests, the samples were collected, isolated, and sent to Fulda, Germany’s Weber & Leucht GmbH laboratory.

The number of substances tested had to be limited due to financial restrictions and quantity of material for research. Pre-tests were carried out to evaluate potential substance classes, mainly with non-destructive spectroscopic technologies. Fourier transform infrared (FTIR) spectroscopy and energy dispersive X-ray (EDX) spectroscopy were used. Also, databases for harmful substances were used for this purpose. Based on the pre-screening results, certain groups of substances were selected, which were classified as conspicuous. This approach made it possible to define the appropriate standards for more precise quantification. The main investigation was carried out in particular for the chromatographic processes with non-destructive extraction (water, methanol).

The contaminants accumulated in the textiles during the conducted experiments are presented in Table 5.

The presented results proved significant differences in the contamination level between the experiments carried out in open and closed spaces. The contaminated samples of Test 2 and 3 contained 1.3–1.8 wt% of soot residues compared to Test 1, where no soot residues were detected. The total PAH values reached of 240 mg/kg in the sample from the PV module fire test carried out in the open space, 364 mg/kg from the PPC sample collected after the PV module fire test in a closed space, and 670 mg/kg in the sample from the car battery fire test in a closed space. Formaldehyde was detected in relatively high concentrations in all contaminated PPC samples and showed the highest concentration in the sample collected after the car battery fire test in a closed space (895 mg/kg).

In terms of metals content, the samples were tested for cobalt and lithium. The results showed that no metals (cobalt and lithium) were detected in either of the PV module fire tests, whereas, in the car battery fire test, both cobalt and lithium were recorded in the samples in the amount of 35 mg/kg for lithium and 24 mg/kg for cobalt.

The contaminated samples were also tested for finding organic phosphoric acid compounds. In the sample taken from PPC contaminated in the open space, such substances were not detected. However, both samples of tests carried out in a closed space showed a content of organic phosphoric acid compounds in the amount of 85 mg/kg for the PV module fire test and 130 mg/kg for the car battery fire test. These organic phosphoric compounds in the contaminated clothing samples may come from burnt components of both the PV modules and the car battery. For the production of PV modules, in addition to selenium, silicon, and germanium, ethylene vinyl acetate (EVA) film are used also as encapsulant materials [44]. EVA foil is flammable, so it may be expected to contain additives that make it flame-resistant. One of the methods of obtaining an EVA polymer with flame retardant properties is the use of ammonium polyphosphate [45]. Phosphorus compounds, including transition metal phosphides are also used in lithium-ion batteries’ anode production [46]. In addition, another phosphorus compound, lithium hexafluorophosphate (LiPF_6_), can be used in fabricating this type of batteries as a component of the liquid electrolyte. Under the conditions of our fire tests, these above mentioned materials could be the source of organophosphorus compounds identified in the samples of the tested clothing.

Oligomer cyclic compound was also detected in all the contaminated PPC samples but surprisingly, the lowest level was observed in the car battery fire test in a closed space (35 mg/kg); then, a higher level was detected in the PV module fire test carried out in the open space (276 mg/kg) and the highest level of this substance was found in the PV module fire test in a closed space (450 mg/kg).

Silicone compounds were only detected in the car battery fire test sample.

## 4. Conclusions and Future Directions

The article presents a review of existing international standards for firefighters’ protective clothing maintenance and handling after contamination. However, these standards do not specify sufficiently precise requirements, especially with regard to the effectiveness of cleaning firefighting clothing and the safety of its re-use. This problem may be getting worse in connection with the emergence of fires that emit more toxic products. These include, among others, the discussed car batteries or PV module fires.

The literature review showed that LCO_2_ cleaning of firefighters’ clothes may be more effective than traditional water cleaning, but it is difficult to be certain that such cleaning method would also be sufficient to remove toxic contaminants after fire events with car batteries or PV modules.

Therefore, in this article it was considered advisable to carry out full-scale pilot fire tests with a car battery and PV modules. During the experiments, toxic smoke was depositing contaminants on the firefighters’ PPC, which was then inspected. It has been proven that even in the event of a fire in the open space, both the PAH and formaldehyde levels were significantly exceeded in relation to the limit values given in the referenced specifications [37,38]. PAH content exceeded the 10 mg/kg limit stated in specifications [37,38] in all scenario sand ranged from 240 mg/kg in Test 1 (in the open space) to 670 mg/kg in Test 3 (in a closed space). It means that even external fires of PV modules cause an intense accumulation of harmful substances in firefighters’ clothing. Formaldehyde was another substance that showed a substantially higher value than the acceptable level of 75 mg/kg indicated for textiles that have direct contact with the skin [37]. It was exceeded six times in Test 1 (470 mg/kg) and almost twelve times in Test 3 (895 mg/kg). Finally, cobalt concentration in clothing samples was recorded with a 24 mg/kg value in Test 3. This level was found to be 24 times greater than the acceptable level of 1 mg/kg [37].

In conclusion, it could be stated that car battery and PV module fires may pose a threat to firefighters health not only during fire events, but also later, when they are under repeated exposure to toxins accumulated on their clothing. Therefore, it is essential to conduct further research on the effectiveness of the available cleaning methods, which the authors of this manuscript plan in the near future. This would enable to develop new guidelines for contaminated firefighters clothing handling and cleaning.

## Figures and Tables

**Table 1:** Limit values for different harmful substances specified by OEKO-Tex (examples) [37].

Substance Type	Substance	Limit Values	
		Direct Contact with the Skin	No Direct Contact to the Skin
Free and partially releasable formaldehyde	Formaldehyde (mg/kg)	75.000	150.000
Extractable heavy metals	Antimony (Sb) (mg/kg)	30.000	30.000
	Arsenic (As) (mg/kg)	0.200	0.200
	Lead (Pb) (mg/kg)	0.200	0.200
	Cadmium (Cd) (mg/kg)	0.100	0.100
	Chromium (Cr) (mg/kg)	1.000	1.000
	Cobalt (Co) (mg/kg)	1.000	1.000
	Copper (Cu) ^1^ (mg/kg)	50.000	50.000
	Nickel (Ni) (mg/kg)	1.000	1.000
	Mercury (Hg) (mg/kg)	0.020	0.020
	Barium (Ba) (mg/kg)	1000.000	1000.000
	Selenium (Se) (mg/kg)	100.000	100.000
	Zinc (Zn) (mg/kg)	750.000	750.000
	Manganese (Mn) (mg/kg)	90.000	90.000
Heavy metals (total content)	Arsenic (As) (mg/kg)	100.000	100.000
	Cadmium (Cd) (mg/kg)	40.000	40.000
	Mercury (Hg) (mg/kg)	0.500	0.500
Phthalates	Each phthalate (w%)	0.010	0.010
	Sum of all phthalates (w%)	0.025	0.025
Other chemical residues	Carcinogenic Arylamines (mg/kg)	20.000	20.000
	Aniline (mg/kg)	20.000	20.000
	Benzene (mg/kg)	1.000	1.000
	Bisphenol A (mg/kg)	100.000	100.000
	Bisphenol B (mg/kg)	1000.000	1000.000
	Diazene-1,2-dicarboxamide (ADCA) (w%)	0.100	0.100
	Phenol (mg/kg)	50.000	50.000
PAHs	Benzo(a)pyrene (mg/kg)	1.000	1.000
	Benzo(e)pyrene (mg/kg)	1.000	1.000
	Benzo(a)anthracene (mg/kg)	1.000	1.000
	Chrysene (mg/kg)	1.000	1.000
	Benzo(b)fluoranthene (mg/kg)	1.000	1.000
	Benzo(j)fluoranthene (mg/kg)	1.000	1.000
	Benzo(k)fluoranthene (mg/kg)	1.000	1.000
	Dibenzo(a,h)anthracene (mg/kg)	1.000	1.000
	Naphthalene (mg/kg)	2.000	2.000
	Sum of 24 PAHs (mg/kg)	10.000	10.000
VOCs and glycols	Methylethylketone (mg/kg)	10.000	10.000
	Ethylbenzene (mg/kg)	10.000	10.000
	Xylene (mg/kg)	10.000	10.000
	Cyclohexanone (mg/kg)	10.000	10.000
	Styrene (mg/kg)	10.000	10.000
	Benzene (mg/kg)	1.000	1.000
	Toluene (mg/kg)	10.000	10.000
Emission of volatiles	Formaldehyde [50-00-0] (mg/cm^3^)	0.100	0.100
	Toluene [108-88-3] (mg/cm^3^)	0.100	0.100
	Styrene [100-42-3] (mg/cm^3^)	0.005	0.005
	Butadiene [106-99-0] (mg/cm^3^)	0.002	0.002
	Vinyl chloride [75-01-4] (mg/cm^3^)	0.002	0.002
	Aromatic hydrocarbons (mg/cm^3^)	0.300	0.300
	Organic volatiles (mg/cm^3^)	0.500	0.500

^1^ no requirement for accessories and yarns made from inorganic materials, respecting the requirements regarding active biological products.

**Table 2:** PAHs limit values specified in the GS specification by the AfPS [38].

Substance	Materials with Long-Term Skin Contact or Repeated Short-Term Skin Contact (mg/kg)	Materials with Short-Term Skin Contact (mg/kg)
Benzo(a)pyrene	<0.5	<1
Benzo(e)pyrene	<0.5	<1
Benzo(a)anthracene	<0.5	<1
Benzo(b)fluoranthene	<0.5	<1
Benzo(j)fluoranthene	<0.5	<1
Benzo(k)fluoranthene	<0.5	<1
Chrysene	<0.5	<1
Dibenzo(a,h)anthracene	<0.5	<1
Benzo(ghi)perylene	<0.5	<1
lndeno(1‚2,3-cd)pyrene	<0.5	<1
Phenanthrene, Pyrene, Anthracene, Fluoranthene, sum	<10	<50
Naphthalene	<2	<10
Sum of 15 PAHs	<10	<50

**Table 3:** The experimental testing conditions.

Test Number	Protective Clothing (Producer, Model)	Composition of the Clothing	Fire Source	Fire Space
Test 1	Scantex, Garda	outer layer: 60% Nomex^®^, 40% viscose FR; membrane: 50% PE, 50% PU FR; thermal insulation: 100% Nomex^®^; liner: 50% Nomex^®^, 50% viscose FR	BAUER PV module BS-310-6MB5 99 × 164 cm	Open
Test 2	Scantex, Garda	outer layer: 60% Nomex^®^, 40% viscose FR; membrane: 50% PE, 50% PU FR; thermal insulation: 100% Nomex^®^; liner: 50% Nomex^®^, 50% viscose FR	BAUER PV module BS-365-M6HBBGG 104 × 177 cm	Closed
Test 3	Ballyclare, Xenon	outer layer: 93% Nomex^®^, 5% Kevlar, 2% antistatic; membrane: 100% Proline PTFE^®^; thermal insulation: 100% Duflot^®^; liner: 50% Kermel, 50% viscose FR	Ni-MH 50 Ah car battery	Closed

**Table 4:** Materials used for pile composition in the experiments.

Material	Width (cm)	Length (cm)	Thickness (cm)	Number (pcs)
OSB	4.5	45.0	2.0	14
OSB	6.0	60.0	2.0	14
Pinewood	4.5	55.0	1.5	4
Kindling cubes	2.1	2.4	1.2	7

**Table 5:** Experimental results (total in all the firefighters clothing layers).

Substance Group	Detection Method/Standard Used	Test 1	Test 2	Test 3
Soot residues, wt%	REM ^1^/EDX/FTIR-ATR ^2^	<0.1	1.3	1.8
Fats/oils/resins, wt%	FTIR-ATR ^2^	<0.01	0.05	<0.01
Total PAHs, mg/kg	DIN EN 17132 ^3^	240	364	670
Formaldehyde ^4^, mg/kg	DIN EN ISO 14184	470	622	895
Lithium, mg/kg	XRF ^5^	<5	<5	35
Cobalt, mg/kg	XRF ^5^	<5	<5	24
Organic phosphoric acid compounds ^6^, mg/kg	LC/MS ^7^	<5	85	130
Oligomer cyclic compound, mg/kg	ISO 15033 ^8^	276	450	35

^1^ Raster electron microscopic analysis; ^2^ attenuated total reflectance; ^3^ total of 18 PAHs mentioned in DIN EN 17132 was measured; limit of quantification was 0.1 mg/kg; ^4^ lower limit of detection was 16 mg/kg; ^5^ X-ray fluorescence analysis; ^6^ two substances in combination were found: tris(2-butoxyethyl)phosphate (TBEP) and tri-n-butyl phosphate (TBP); ^7^ liquid chromatography—mass spectrometry; ^8^ lower limit of detection was 5 mg/kg.

## Data Availability

Not applicable.

## Abbreviations and Short Forms

AfPS	Ausschuss für Produktsicherheit (Committee for Product Safety)
AMD	Amendment
BS	British Standard
CBRN	Chemical, Biological, Radiological or Nuclear
DIN	Deutsches Institut für Normung (German institute for Standardization)
ECHA	European Chemicals Agency
EDX	Energy Dispersive X-ray Spectroscopy
EV	Electric Vehicles
EN	European Norm
EVA	Ethylene Vinyl Acetate
FR	Fire Resistant
FTIR-ATR	Fourier Transform Infrared—Attenuated Total Reflectance
HazMat	Hazardous Materials
ISO	International Organization for Standardization
LC/MS	Liquid Chromatography—Mass Spectrometry
LCO_2_	Liquid CO_2_
NFPA	National Fire Protection Association
OEKO-TEX^®^	Registered trade mark, representing the product labels and company certifications issued and other services provided by the International Association for Research and Testing in the Field of Textile and Leather Ecology
OETI	Institut fuer Oekologie, Technik und Innovation GmbH (Institute for Ecology, Technology and Innovation)
OSB	Oriented Strand Board
PE	Polyethylene
PPC	Personal Protective Clothing
PPE	Personal Protective Equipment
PU	Polyurethane
PV	Photovoltaic
PAHs	Polycyclic Aromatic Hydrocarbons
REACH	Registration, Evaluation, Authorisation and Restriction of Chemicals—European Union regulation
REM	Raster Electron Microscopic Analysis
VOCs	Volatile Organic Compounds
XRF	X-ray Fluorescence Analysis
